# Preparation of Electrospun Nanocomposite Nanofibers of Polyaniline/Poly(methyl methacrylate) with Amino-Functionalized Graphene

**DOI:** 10.3390/polym9090453

**Published:** 2017-09-16

**Authors:** Hanan Abdali, Abdellah Ajji

**Affiliations:** 1 CREPEC, Department of Chemical Engineering, Polytechnique Montréal, P.O. Box 6079, Station Centre-Ville, Montreal, QC H3C 3A7, Canada; hanan.abdali@polymtl.ca; 2 Ministry of Higher Education, P.O. Box 225085, Riyadh 11153, Saudi Arabia

**Keywords:** electrospun nanofibers, electrospinning, polyaniline, nanocomposites, amino-functionalized graphene

## Abstract

In this paper we report upon the preparation and characterization of electrospun nanofibers of doped polyaniline (PANI)/poly(methyl methacrylate) (PMMA)/amino-functionalized graphene (Am-rGO) by electrospinning technique. The successful functionalization of rGO with amino groups is examined by Fourier transforms infrared (FTIR), X-ray photoelectron spectroscopy (XPS) and Raman microspectrometer. The strong electric field enables the liquid jet to be ejected faster and also contributes to the improved thermal and morphological homogeneity of PANI/PMMA/Am-rGO. This results in a decrease in the average diameter of the produced fibers and shows that these fibers can find promising uses in many applications such as sensors, flexible electronics, etc.

## 1. Introduction

Electrospinning is an efficient, relatively simple and low-cost process used to produce continuous nanofibers on a large scale, where the fiber diameter can be adjusted from nanometers to microns by applying a high voltage to a polymer solution from a micro-syringe pump [1,2,3,4,5]. Polymer nanofibers produced via electrospinning have specific surface areas approximately one to two orders of magnitude larger than flat films, making them the most promising candidates for applications in filtrations, engineering tissue scaffolds, wound healing, release control, energy storage and sensors [5,6,7,8].

Polyaniline (PANI) is one of the most conductive polymers, that has been used in many electronic, optical and electrochemical applications, due to its low cost, good environmental stability, redox reversibility, and electrical conductivity [9,10]. However, processing PANI into nanofibers by using the electrospinning is a challenge, mainly due to its rigid backbone that is related to its high degree of aromaticity making the elastic properties of the solution insufficient for electrospinning [11,12]. In this regard, non-conductive hosting polymers such as poly(methyl methacrylate) (PMMA), is blended to assist polyaniline to form composite fibers [13,14]. Consequently, the nanofibers of PANI have garnered much interest because of their properties as candidates for chemical sensors [15], light-emitting and electronic devices [16]. Yet, some disadvantages such as poor mechanical properties do exist, although combining PANI with carbon materials reinforces its stability and enhances some of its properties, such as capacitance [17,18].

Graphene is a potential nanofiller that can efficiently enhance the mechanical, thermal and electrical properties of polymer-based nanocomposites at a very low loading, useful for various novel applications due to its high thermal conductivity, superior mechanical strength, high specific surface area, excellent mobility of charge carriers and high chemical stability [19,20,21]. However, the homogenous dispersion of graphene in a polymer matrix is a necessary feature when it is used as a nanofiller. Graphene is predisposed to agglomerate because of its hydrophobic nature, high surface energy, and intrinsic van der Waals forces preventing its uniform distribution in the polymer matrix [21,22,23]. This reduces its beneficial effects and therefore dispersing the graphene in an electrospinning solution is an important step in forming the nanofibers [21]. The problems can be overcome by functionalizing the graphene. This procedure provides multiple bonding sites to the resin matrix where the remarkable properties of graphene can be successfully transferred to a polymer composite [23,24,25]. Amine groups are attributed with high reactivity enabling them to react with other chemical groups easily and providing a favorable approach in the preparation and applications of graphene/polymer nanocomposites [23]. As the Nitrogen atom in amine is more nucleophilic than the oxygen atom, it can be expected that substituting graphene or graphene oxide with amine will increase the nucleophilic properties of graphene. Consequently, interfacial binding can result between graphene and the materials of interest. These interactions will improve the performance and functionality of the intended applications of graphene [23,24]. Former studies about the functionalization of graphene with amine groups have indicated that it could be a promising strategy to improve the electrical conductivity of graphene, due to the electron donating effect of amine groups [23]. 

Herein, the ethylenediamine (NH_2_–(CH_2_)_2_–NH_2_) was utilized to functionalize graphene surfaces, which produced stitched graphene owing to the presence of two amine (–NH_2_) functionalities on both sides of the ethylene moiety [24]. Therefore, due to the intriguing properties of graphene and the advantages of PANI, composites of graphene and PANI fibers are eminently suitable for many applications such as organic photovoltaics, supercapacitors and resistance-based sensors. In this article, the preparation of electrospun fiber mats of doped polyaniline/poly(methyl methacrylate)/amine-functionalized graphene using the electrospinning process is studied. Literature has reported on graphene/polyaniline composites [26,27,28] but, in this study, for the first time, amino-functionalized graphene/polyaniline nanofibers are investigated using the electrospinning process. 

More specifically, the objectives of this study were to identify and detail the primary materials and process factors necessary to produce amino-graphene/polyaniline nanofibers using the electrospinning process. The details of the amine functionalization of graphene are presented and the morphology and the thermal stability of the PANI/PMMA/Am-rGO nanofibers are investigated.

## 2. Materials and Methods

### 2.1. Materials

Graphene oxide (GO), poly(methyl methacrylate) (PMMA) *M*w ~ 996,000 g·mol^−1^, polyaniline (PANI, emeraldine base) *M*w ~ 100,000 g·mol^−1^, camphor-10-sulfonic acid (HCSA, 98%), *N*,*N* dimethylformamide (DMF, 99.8%), chloroform (CHCl_3_, ≥99%), ethylenediamine (EDA, ≥99%), were all purchased from Sigma-Aldrich, (Oakville, ON, Canada ). Deionized (DI) water was used for all the experiments.

### 2.2. Reduction of Graphene Oxide to Graphene

Commercial graphene oxide (GO) was reduced by thermal annealing treatment [29]. First, the GO powder was heated in a tubular quartz furnace (High Temperature Tube Furnace (HTF), GSL-1300-40X, MTI Corporation, CA, USA) from room temperature to 400 °C at the rate of 5 °C·min^−1^, and kept at 400 °C for 30 min under an argon (Ar) gas flow of 80 mL·min^−1^; secondly, the temperature was increased from 400 to 650 °C at a rate of 5 °C·min^−1^, and kept at 650 °C for 30 min under an Ar gas flow of 80 mL·min^−1^. Finally, the reduced GO samples were naturally cooled to room temperature without argon.

### 2.3. Surface Modification of Graphene with Amines

The rGO made in the previous step, where 150 mg of rGO was mixed with 10 mL of EDA, in a vessel under vigorous stirring. The reaction was continued for 24 h under reflux at 80 °C. Afterwards, the aminated-rGO (Am-rGO) was centrifuged at 10,000 rpm for 1 h and was thoroughly washed with deionized water, filtered, and dried in a vacuum oven at 80 °C for 24 h [23].

### 2.4. Preparation of the PANI/PMMA/Am-rGO Solution

10 mg of the Am-rGO was dispersed in 2.96 g of DMF by sonication for 1 h. 100 mg of PANI was mixed with 130 mg of HCSA to dope it and dissolving it in 14.78 g of chloroform. The solution was stirred constantly for 6 h and subsequently filtered using Whatman Puradisk PTFE syringe filter (pore size—0.45 μm, GE Healthcare, Buckinghamshire, UK) to remove the particulate matter. Then, the Am-rGO solution was mixed with PANI solution and subsequently 85 mg of PMMA (as a carrier polymer) was added to the solution and stirred for 24 h to form the solution for electrospinning (see Table 1). The PANI/PMMA solution was similarly prepared without the addition of functionalized graphene for comparative analysis.

### 2.5. Electrospinning Setup

**Figure 1:**
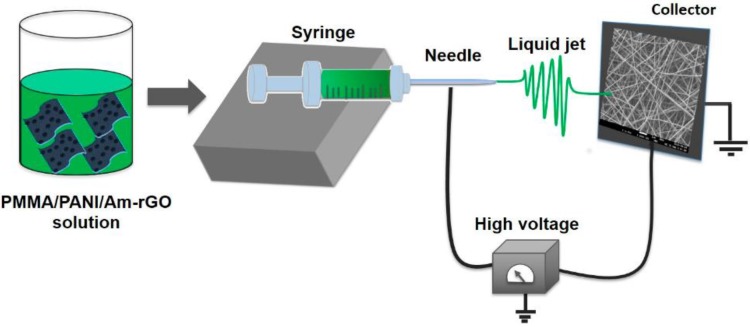
Schematic illustration of the electrospinning setup.

Figure 1 shows the schematic diagram of the fabrication of graphene-polymer nanofiber composite by electrospinning. The homogeneous dispersed solutions were electrospun using a homemade horizontal set up containing a programmable micro-syringe pump (Harvard Apparatus, PHD 2000, Holliston, MA, USA) and a variable high DC voltage power supply (ES60P-5W Gamma High Voltage Research Inc, Omaha Beach, FL, USA). Parameters such as the flow rate, voltage and distance were harnessed at peak efficiency to obtain the desired nanofibers with the least beading to perform the subsequent experiments. The PANI/PMMA/Am-rGO solution was loaded into a 3 mL syringe with Luer-Lock connection to an 18-gauge blunt tip needle (Cadence Science, Cranston, RI, USA). The syringe was mounted on the pump with a grip and grounded by use of an alligator clip. The applied voltage was in the range of 18–20 kV between the needle tip and the collector. A syringe pump was utilized to control the flow rate of the solution which was 0.3 mL/h and the distance between the needle and the collector was 15 cm. The spun nanofiber mats were collected on an aluminum foil attached to a stationary collector plate. All experiments were conducted in a chamber at a relative humidity of 19–25%. 

### 2.6. Characterization of Amino Functionalized Graphene 

In order to ensure the presence of functional groups that should be present from the successful reduction of GO to rGO and functionalization of rGO with amino group, the Fourier transform infrared (FTIR) spectroscopy analysis was undertaken using Perkin Elmer 65 FTIR-ATR instrument (PerkinElmer, Woodbridge, ON, Canada). A total of 128 scans were accumulated for signal averaging of each IR spectral measurement with a 4 cm^−1^ resolution. The spectra of the samples were recorded over a wavenumber range of 4000–650 cm^−1^. Raman microspectrometer and the electron diffraction (SAED) patterns were utilized to investigate the structural changes of GO, rGO, and Am-rGO. Characterization of graphene specimens were performed by Raman microspectrometer with a Renishaw InVia Raman microscope (Renishaw, Mississauga, ON, Canada) at an excitation laser wavelength of 514 nm. Raman spectroscopy is a powerful non-destructive tool for studying disorder and defects in crystal structure, it is often used to characterize microstructure of carbon materials. All specimens were deposited on glass slides in powder form without any solvent. For SAED patterns characterization, the graphene specimens were scooped onto a transmission electron microscopy (TEM) copper grid with supporting carbon film (CF400-Cu, Electron Microscopy Sciences) directly. The dispersion characteristics of rGO and Am-rGO were measured by ultraviolet visible (UV–Vis) spectrophotometer at ambient temperature utilizing Infinite 200 PRO (Tecan, Männedorf, Switzerland) cuvette reader. The chemical composition of the samples were determined by X-ray photoelectron spectroscopic (XPS) analysis using a VG Scientific ESCALAB 3 MK II X-ray photoelectron spectrometer (VG Escalab 3 Mk, East Grinstead, England) using an Mg Kα source (15 kV, 20 mA).

### 2.7. Characterization of the PMMA/PANI/Am-rGO Nanofibers 

The scanning electron microscope (SEM, JSM-7600TFE, FEG-SEM, Calgary, AB, Canada) at an operational voltage of 2 kV was used to study the morphology of electrospun fibers. Fiber diameters were calculated using Image-Pro Plus® software by taking an average of about 300 fibers. To confirm the presence of graphene sheets in the nanofibers of PANI/PMMA/Am-rGO, transmission electron microscopy (TEM, JEOL, JEM 2100F, JEOL, Pleasanton, CA, USA) was used. For TEM observation, fibers were directly deposited onto a TEM copper grid with supporting carbon film (CF400-Cu, Electron Microscopy Sciences). Thermogravimetric analysis (TGA) was conducted under nitrogen atmosphere using Q5000 TGA (TA Instruments, New Castle, DE, USA) in the temperature range of 20–900 °C, with a heating ramp of 10 °C·min^−1^.

## 3. Results and Discussion

### 3.1. Morphology and Structure Analysis of Am-rGO

**Figure 2:**
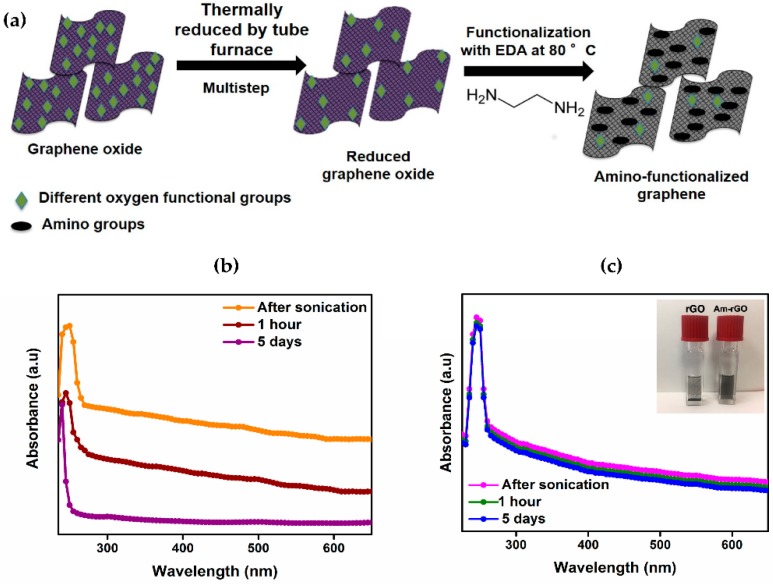
(**a**) Schematic illustration of the non-covalent functionalization of graphene surfaces with amino groups. Time evolution of UV-Vis absorption spectra of (**b**) rGO and (**c**) Am-rGO dispersed in CHCl_3_/DMF (inset shows photograph of GO, rGO and Am-rGO).

The UV–Vis spectrum of rGO suspension showed an absorption peak at around 265 nm. This observation confirms the formation of C=C conjugated graphene structure after the thermal reduction process (see Figure 2b) [24]. The UV–Vis spectroscopy was used also for monitoring the stability of the rGO and Am-rGO in mixture of CHCl_3_/DMF (5:1). As shown in Figure 2b,c, there is a very slight decrease in the absorbance spectra of Am-rGO over five days in comparison with rGO, indicating a good stability of the Am-rGO dispersion. Visually, dispersion of Am-rGO is more stable, whereas in comparison with dispersions of rGO in the same solution, which means that the dispersion of Am-rGO is greatly improved within CHCl_3_/DMF in the presence of amino groups (in the inset of Figure 2c). 

**Figure 3:**
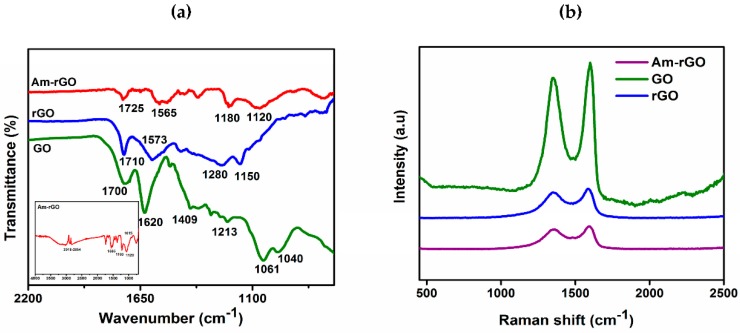
(**a)** FTIR and (**b**) Raman spectra of GO, rGO and Am-rGO.

FTIR spectroscopy was performed on GO, rGO and Am-rGO in order to ensure the presence of functional groups that should be present from the successful reduction of GO to rGO and functionalization of rGO with amino groups (see Figure 2a). Different oxygen containing functional groups were observed on the GO spectrum bands as shown in Figure 3a. The C=O stretching vibrations in the carboxyl groups at 1700 cm^−1^; the C–OH deformation from the hydroxyl groups attached to the aromatic graphene network at 1409 cm^−1^; the C–O (hydroxyl) stretching at 1601 cm^−1^; the C–O (epoxy) stretching at 1040 cm^−1^ and the C–O (phenolic) stretching at 1213 cm^−1^ [18,30]. The band at 1620 cm^−1^ is ascribed to the skeletal vibration of unoxidized sp^2^ graphitic domains. After the thermal reduction of GO, the skeletal vibration of sp^2^ graphitic domain still remains shifts to 1573 cm^−1^. Besides, some residual presence of bands at 1710, 1150, and 1280 cm^−1^ were detected providing evidence of the different types of oxygen functionalities in the rGO and their decreases in intensity and others vanished due to thermal reduction [18,30]. In the spectrum of Am-rGO, the N–H deformation peaks at 1565 cm^−1^; the C–N stretching vibrations at 1180 and 1120 cm^−1^; and the C=O stretching vibrations of carboxyl group at 1725 cm^−1^. Furthermore, the Am-rGO has peaks of the C–H stretch of alkyl chain at 2918 and 2854 cm^−1^; and the C–O stretching in hydroxyl groups at 1015 cm^−1^. The FTIR spectroscopy results indicate that the chemically functionalized graphene (Am-rGO) was successfully synthesized. Similar results for functionalization of graphene with amino groups have been previously reported in literature [23,30].

Raman spectra was employed to analysis the graphitic structures of of GO, rGO, Am-rGO, as shown in Figure 3b. The G band is derived from stretching the C–C bond, and is usual for all sp^2^ carbon forms system, and it is obtained from the first order Raman scattering [31,32], and the D band is due to disordered carbon atoms [31,32]. As expected, the GO revealed an intensive G band at 1600 cm^−1^ owing to the oxygenation of graphite, which results in the formation of sp^3^ while, the D band is presented at 1350 cm^−1^ because of the reduction in size of the sp^2^ domains by the creation of defects, and distortions during oxidation [32,33]. Moreover, in rGO and Am-rGO, the G band were shifted to lower wavenumber exhibited at 1587 and 1595 cm^−1^ and the D band positions remained unchanged at 1350 cm^−1^, respectively. The G band appears in lower frequency due to the increased number of sp^2^ carbon atoms following reduction of GO. The intensity ratio of D and G band *I*_D_/*I*_G_ is slightly increased from (0.89) in GO to (0.93) in rGO, demonstrating a decrease in the size of the in-plane sp^2^ domains after reduction, and can be explained that the thermal redaction creates many new graphitic domains, that are more numerous in number and smaller in size [32,33,34]. Whereas, the Am-rGO showed a higher I_D_/I_G_ intensity ratio (0.97) than the rGO, which is attributed to the formation of the chemical bond between amino groups and basal planes of the rGO. This corresponds to other results reported for functional graphene [34,35,36].

**Figure 4:**
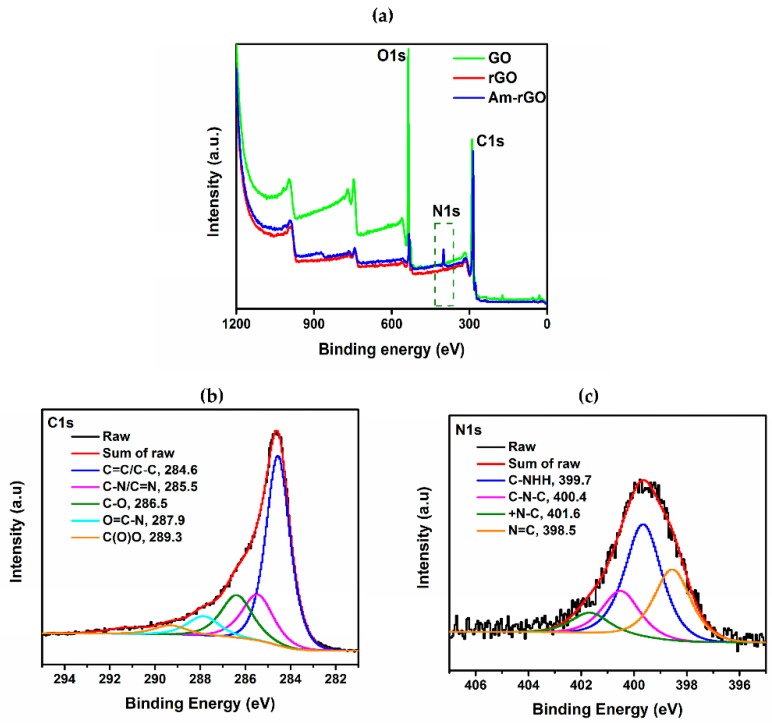
(**a**) XPS survey of GO, rGO and Am-rGO. High resolution XPS spectra of (**b**) C1s and (**c**) N1s of Am-rGO.

The XPS was also applied as an effective tool to characterize the presence of different elements such as carbon, oxygen and nitrogen in GO, rGO and Am-rGO samples. Table 2 shows that the elemental analysis of GO, rGO and Am-rGO. The results confirm the successful functionalization of rGO with amino groups. The increase in C/O atomic ratio in Am-rGO indicates that EDA can successfully functionalized graphene sheets. The presence of N containing groups in Am-rGO can also be demonstrated from its XPS spectrum, where three peaks corresponding to N, C and O elements can be clearly visualized. As shown in Figure 4a, only the carbon (C1s at 284.8 eV) and oxygen (O1s at 531.2 eV) appeared in the wide-scan spectrum in the GO, rGO and Am-rGO. After the functionalization of rGO with amino groups, as expected, the nitrogen (N1s at 400.1 eV) was clearly evident in the wide-scan spectrum in the Am-rGO [30,37]. The appearance of the N1s peak and the greatly decreased intensity of the O1s peak in the XPS spectrum of Am-rGO indicate the efficient displacement of oxygen moieties by amino groups during the chemical amination of rGO [30,37]. Peak fitting of C1s and N1s high resolution C1s and N1s XPS spectrum reveals the diverse carbon and nitrogen components in the Am-rGO framework. As shown in Figure 4b, carbon atoms exists in different functional groups: at 284.6 (C–C/C=C), 285.5 (C–N/C=N), 286.5 (C–O), 287.9 (O=C–N) and 289.3 eV (O–C=O). The amination process led to the formation of (N=C) at 398.5 eV, (C–NH_2_) at 399.7 eV, (C–N–C) at 400.4 eV, and (C–N^+^–C) at 401.6 eV (see Figure 4c) [29,30]. As it can be seen in Figure 4c, the most intense peak is assigned to the C–NH_2_, indicating that amino functionalized-rGO was successfully prepared. These XPS results were consistent with other studies presented in the literature [30,37].

**Figure 5:**
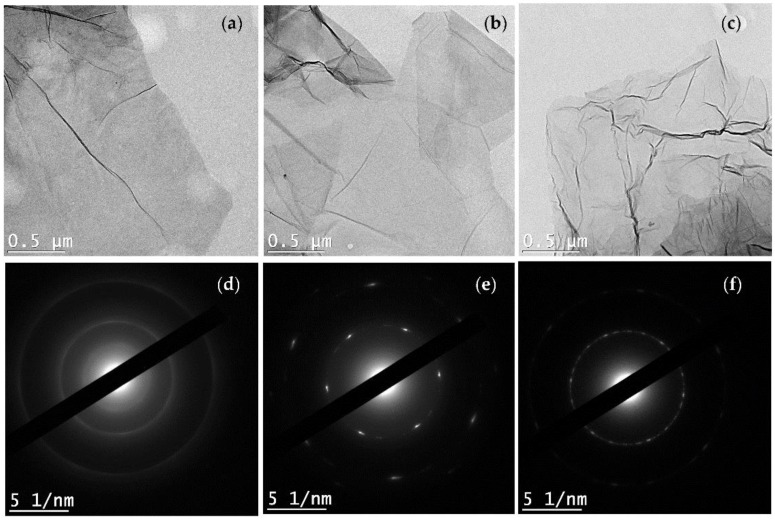
TEM images of (**a**) GO; (**b**) rGO and (**c**) Am-rGO with (**d**–**f**), respective SAED pattern.

The morphology and microstructure of the GO, rGO and Am-rGO and the electron diffraction (SAED) patterns in the selected area were analyzed by TEM (see Figure 5a–f). The TEM images of the GO, rGO and Am-rGO in Figure 5a–c, respectively, clearly show the presence of wrinkles, ripples and scrolls in the GO, rGO and Am-rGO indicating the occurrence of few-layered graphene sheets [38,39]. The SAED patterns of GO, rGO and Am-rGO (Figure 5d–f) were compared in order to understand the successful reduction and fictionalization with amino groups. Only diffraction rings are found in the SAED pattern of the GO, demonstrating the disordered structure of GO, while the diffraction spots in the rGO confirm the crystalline structure obtained after the thermal reduction of GO [38,39,40], as shown in Figure 5d–e. Moreover, owing to the addition of amino functional groups, the bright spots were not fully restored into the hexagonal graphene framework [40] (see Figure 5f). These results show that functionalization caused less damage to the graphene structure.

### 3.2. Nanofibers Morphology

**Figure 6:**
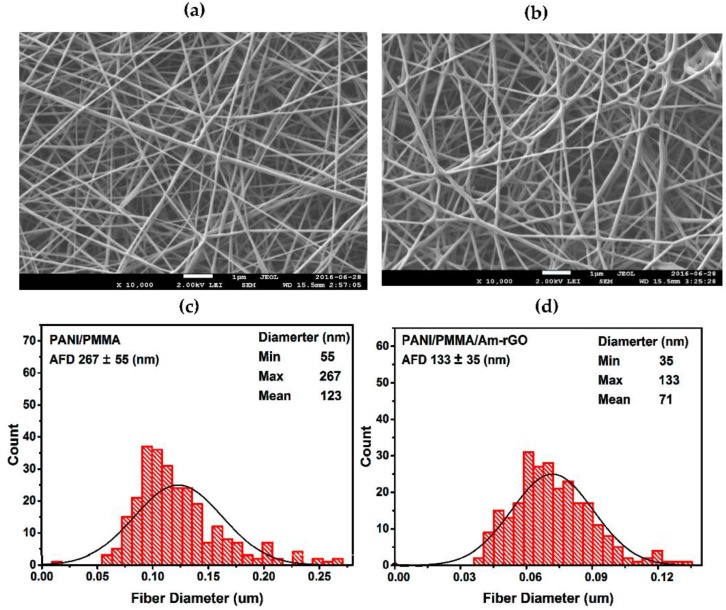
(**a**,**c**) SEM micrograph of the PANI/PMMA and PANI/PMMA/Am-rGO nanofibers, respectively; (**b**,**d**) the distribution of dimeters of the PANI/PMMA and PANI/PMMA/Am-rGO nanofibers, respectively.

SEM was used to characterize the fabricated PANI/PMMA and PANI/PMMA/Am-rGO nanofibers (see Figure 6a–d). It is observed that the surface of nanofibers are relatively smooth and randomly oriented forming an web-like pattern [18,41]. Yet, owing to the instability of the liquid jet, beads can be observed in the image of PANI/PMMA/Am-rGO. Additionally, the average diameter before adding Am-rGO were in the range 267 ± 55 nm and after adding Am-rGO, the average diameter decreased to the range 133 ± 35 nm. This decrease in the average fiber diameter of PANI/PMMA/Am-rGO in comparison to PANI/PMMA is due to the presence of graphene sheets in the fibers. This could be attributed to the electrical conductivity in the starting solution enhanced by the presence of the graphene where the more conductive the solution, the better the chance of getting thinner fibers [18,41]. Therefore, incorporating graphene into PANI/PMMA solution enhances the conductivity of the solution to be electrospun and as a result of this improved conductivity, the produced fibers become thinner compared with fibers produced from PANI/PMMA solution [18,41].

**Figure 7:**
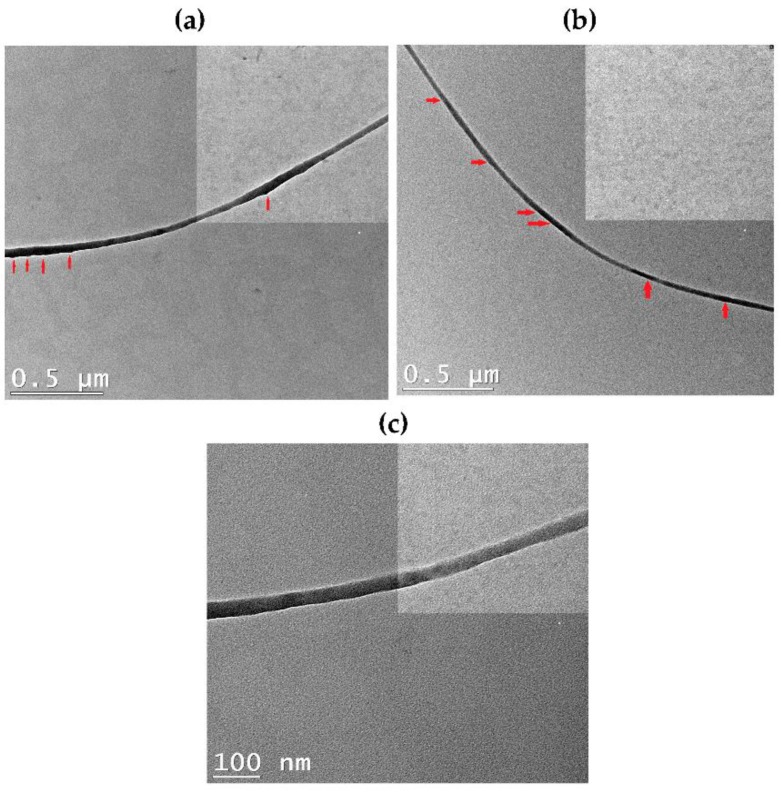
(**a**–**c**) High-resolution TEM images of PANI/PMMA/Am-rGO nanofibers at different magnification.

TEM was conducted to confirm the presence of the graphene filler in the nanofibers. Figure 7a shows that some of the incorporated Am-rGO are randomly embedded in the sidewall of PANI/PMMA nanofibers. Moreover, Figure 7b–c illustrate that along the nanofibers some dark scattering spots could be observed; these aligned dark dots corresponded to graphene flakes in the PANI/PMMA nanofibers. These figures clearly show the individual graphene sheets dispersed in the PANI/PMMA matrix without aggregation, because the lateral size of graphene (a few 100 nm to a few µm) is comparable to the fiber diameter [37,38,39,40,41]. 

### 3.3. Thermal Stability

**Figure 8:**
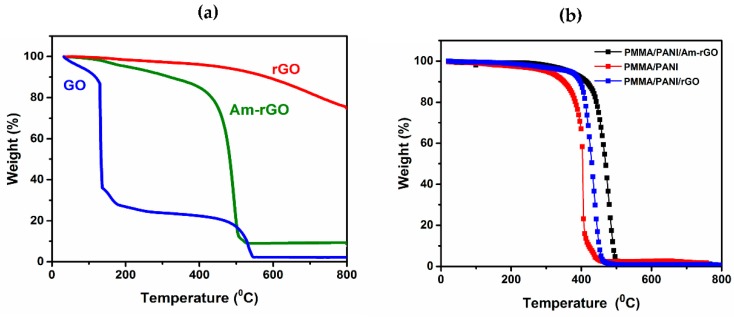
TGA curves (**a**) of GO, rGO and Am-rGO; and (**b**) for electrospun PMMA/PANI/Am-rGO, PMMA/PANI/rGO and PMMA/PANI nanofibers.

TGA was performed to observe the thermal stability of the rGO, Am-rGO and electrospun PANI/PMMA and PANI/PMMA/Am-rGO nanofiber mat (see Figure 8a,b), respectively, under N_2_ atmosphere in temperature range between 20 and 900 °C, with a heating ramp of 10 °C·min^−1^. GO is thermally unstable resulting in three stages of weight loss. In the first stage, a rapid weight loss occurs at about 173 °C, mostly attributed to the removal of the trapped water molecules and epoxy oxygen functional groups. The second stage occurs at 515 °C which can be attributed to the removal of phenolic groups and decomposition of sp^3^ hybridized carbon atoms located at the defect site of GO. These results were congruent with other studies presented in literature [30,37]. On the other hand, the rGO was the most thermally stable, there is almost no weight loss below 600 °C, demonstrating the effective reduction and removal of oxygen functional groups. Comparing with GO, the Am-rGO exhibits good thermal stability. The thermal stability increases and major weight loss starts at temperatures of about 449 °C. This can be ascribed to the decomposition of amino-carbons, which is similar to the previously reported results for functionalization of graphene with amino groups [30,37]. Therefore, the larger thermal stability compared with GO and the greater mass lost compared with rGO indicates the efficient displacement of oxygen moieties by amino groups during the chemical amination of rGO [37].

Incorporating a small amount of Am-rGO in PANI nanofibers improves its thermal stability. As shown in Figure 8b, the thermal degradation temperature of PMMA/PANI/Am-rGO nanofibers increased to ~441 °C, a magnitude higher than that of the PMMA/PANI samples at ~348 °C, accredited to the presence of interfacial bonding [18,41]. In comparison to PMMA/PANI/Am-rGO, the thermal degradation temperature of the PMMA/PANI/rGO was presented around 420 °C, and corroborating the strong interaction exists between the PANI and the Am-rGO. This thermal reinforcement of the electrospun PANI nanofibers by Am-rGO (nano-carbon) fillers is very important in many different technological applications such as those mentioned in the introduction.

## 4. Conclusions

In this study, Am-rGO was successfully prepared and electrospun nanofibers of PANI/PMMA with amino-functionalized graphene were prepared by a simple electrospinning technique. The FTIR, XPS and Raman spectroscopy analysis confirmed the successful functionalization of the graphene with amino groups, while the TEM observation demonstrated that the addition of amino-functional groups to graphene generated less damage to the graphitic structure of the graphene. SEM micrographs indicated the formation of PANI/PMMA/Am-rGO nanofibers with a diameter ranging between 35 and 133 nm, with a general uniform thickness along the nanofibers. TGA measurements show an improvement of the thermal stability of the PANI in the presence of graphene. In conclusion, the resulting non-woven porous mats with amino-functionalized graphene result in electrospun nanofibers that can be used in future technological applications in various fields. 

## Figures and Tables

**Table 1:** Composition of electrospun PANI/PMMA/Am-rGO and PANI/PMMA solutions.

PANI (mg)	HCSA (mg)	Am-rGO (mg)	CHCl_3_ (g)	DMF (g)	PANI:PMMA (wt %)	Am-rGO:PANI (wt %)
100	130	10	14.78	2.96	54.05	9.09
100	130	-	14.78	2.96	54.05	-

**Table 2:** Elemental composition of GO, rGO and Am-rGO samples extracted based on the XPS results.

Elements	Relative atomic percent (%)			C/O Ratio
	C	O	N	
**GO**	66.4	32.5	0.3	2.0
**rGO**	86.1	12.7	0.3	6.7
**Am-rGO**	83.2	10.2	6.6	8.2
